# Antioxidant and Anti-Diabetic Properties of Olive (*Olea europaea*) Leaf Extracts: In Vitro and In Vivo Evaluation

**DOI:** 10.3390/antiox12061275

**Published:** 2023-06-14

**Authors:** Hanem M. M. Mansour, Ashraf A. Zeitoun, Hagar S. Abd-Rabou, Hesham Ali El Enshasy, Daniel Joe Dailin, Mohamed A. A. Zeitoun, Sobhy A. El-Sohaimy

**Affiliations:** 1 Department of Food Technology, Arid Lands Cultivation Research Institute, City of Scientific Research and Technological Applications, Alexandria 21934, Egypt; hmahmoud@srtacity.sci.eg (H.M.M.M.);; 2 Department of Food Science, Faculty of Agriculture (Saba Basha), Alexandria University, Alexandria 21934, Egypt; prof.dr.ashrafabdelmoneimzaitoun@alexu.edu.eg (A.A.Z.);; 3 Institute of Bioproduct Development (IBD), Universiti Teknologi Malaysia (UTM), Skudai 81310, Malaysia; henshasy@ibd.utm.my (H.A.E.E.);; 4 School of Chemical and Energy Engineering, Universiti Teknologi Malaysia (UTM), Skudai 81310, Malaysia; 5 Genertic Engineering and Biotechnology Research Institute, City of Scientific Research and Technology Applications, Alexandria 21934, Egypt; 6 Department of Technology and Organization of Public Catering, South Ural State University, 454080 Chelyabinsk, Russia

**Keywords:** olive (*Olea europaea*) leaf extracts, HPLC analysis, antioxidant activity, hemolytic activity, α-glucose oxidase inhibitor, antidiabetic

## Abstract

**(1) Objective:** The main objective of the current study was to evaluate in vitro and in vivo an antioxidant property of three genotypes of olive leaf extract (OLE) (picual, tofahi and shemlali), and furthermore to assess potential activity in the treatment and/or prevention of diabetes mellitus type II and related implications. **(2) Methodology:** Antioxidant activity was determined by using three different methods (DDPH assay, reducing power and nitric acid scavenging activity). In vitro α-glucosidase inhibitory activity and hemolytic protective activity were assessed for the OLE. Five groups of male rats were used in in vivo experiment for evaluating the antidiabetic potential of OLE. **(3) Results:** The genotypes of the extracts of the three olive leaves exhibited meaningful phenolic and flavonoids content with superiority for picual extract (114.79 ± 4.19 µg GAE/g and 58.69 ± 1.03 µg CE/g, respectively). All three genotypes of olive leaves demonstrated significant antioxidant activity when using DPPH, reducing power and nitric oxide scavenging activity with IC_50_ ranging from 55.82 ± 0.13 to 19.03 ± 0.13 μg/mL. OLE showed a significant α-glucosidase inhibition activity and dose-dependent protection from hemolysis. In vivo experimentation revealed that the administration of OLE alone and the combination of OLE+ metformin clearly restored the blood glucose and glycated hemoglobin, lipid parameters and liver enzymes to the normal level. The histological examination revealed that the OLE and its combination with metformin successfully repaired the liver, kidneys and pancreatic tissues to bring them close to the normal status and maintain their functionality. **(4) Conclusion:** Finally, it can be concluded that the OLE and its combination with metformin is a promising treatment for diabetes mellitus type 2 due to their antioxidant activity, which emphasizes the potential use of OLE alone or as an adjuvant agent in the treatment protocol of diabetes mellitus type II.

## 1. Introduction

*Olea europaea* L. is an evergreen tree belonging to the Oleaceae family and cultivated on a broad spectrum all over the world, especially in the Mediterranean basin countries. Olive trees can be found in three areas in Egypt: North Sinai, the Alexandria–Cairo Road and Siwa. Egypt is among the top 10 countries in olive production worldwide, producing 332,321 tons of olives annually. Egypt has a total cultivation area of 100,000 ha, almost 1% of the world’s total [1]. The olive industry produces a huge amount of by-products that can have a negative impact on the environment if not treated properly and correctly [2]. One of the major olive industry by-products are olive leaves, produced by the pruning and harvesting of olive trees. Olive leaves obtained as a by-product during the harvesting or fabrication process of olive fruits contain considerable bioactive compounds which exhibit many potential health benefits such as antioxidant activity anti-HIV properties, anti-proliferative and apoptotic effects, protective effects against human leukemia and lipid-lowering activity [3,4]. Olive leaf extracts have received special interest from researchers all over the world for their therapeutic applications. These extracts have different classes of biophenols, including phenolic acids, phenolic alcohols (hydroxytyrosol and tyrosol), flavonoids (luteolin 7-O-glucoside, rutin, apigenin 7-O-glucoside, luteolin 4-O-glucoside) and secoiridoids (oleuropein) [5,6]. A large number of bioactive compounds such as phytochemicals can be found in olive leaves including oleuropein as a main phenolic molecule. The antioxidant properties of oleuropein have been demonstrated in several previous studies, which may explain their biological and pharmacological properties [7,8]. Olive leaves which contain rich phenolic compounds are marketed as olive leaves for tea or food supplements for pharmaceutical purposes. The phytochemical studies conducted so far have demonstrated that olive leaves contain secoiridoids, most importantly oleuropein, flavonoids such as luteolin 7-O-glucoside (L7G) and apigenin-7-O-glucoside, as well as some triterpenoids and lignan derivatives [9]. Glucose is indispensable to human health because it is a fundamental source of energy for the muscles, tissues and brain cells. Diabetes mellitus is a disease that affects the metabolism of glucose in the body, resulting in an increase in the blood glucose level (hyperglycemia). When insulin is not produced enough by the body or the cells fail to respond to insulin, too much blood sugar remains in the bloodstream, resulting in diabetes mellitus. Chronic diabetes (includes type 1 and type 2) might cause many severe health implications, such as chronic kidney disease, cardiovascular diseases, stroke and vision loss. Nowadays, pharmaceutical companies give a lot of attention to plant-based bioactive compounds for the development of innovative drugs and food supplements to control some disease such as diabetes mellites, cancer and cholesterol and cardiovascular problems with minimum side effect [10]. Olive leaf extract is the best source of natural products among other plants and herbs for controlling the level of blood sugar for diabetic patients [11,12]. Many people prefer to take a combination of herbs and prescribed modern medications to receive the best antidiabetic effect due to the potential synergistic interaction between them [13]. Herbs and drugs may interact negatively or positively in terms of their pharmacological or toxicological behavior. The nutraceutical effect of olive leaf extract comes from its ability to modulate plasma glucose levels through insulin release. Other bioactive compounds are capable of influencing postprandial glycaemia by reducing insulin resistance [14]. Furthermore, randomized clinical trials have emphasized the ability of olive leaf extract to improve glucose homeostasis in diabetic patients [15]. Therefore, the present work was focused on the in vitro and in vivo evaluation of the potential antioxidant and anti-diabetic effect of olive leaf extract, which will add value to the olive leaf by-products and allow their use in the development of health-promoting food supplements. 

## 2. Materials and Methods

### 2.1. Plant Materials

Three Egyptian genotypes of the *Olea europaea* plant were used in this study. Picual (P), shemlali (S) and tofahi (T) cultivars were selected from the experimental farm of the City of Scientific Research and Technological Applications, Alexandria, Egypt, in January 2019. The collected olive leaves were washed, packed in polyethylene bags and then stored at −80 °C for their next use.

### 2.2. Extraction of Phenolic Compounds 

A household steam cooker was used to blanch olive leaves at 90 °C for two minutes, then they were immediately cooled down with cold water at 17 °C to enhance the phytochemicals’ recovery. Excess water was removed with an absorbent paper and the leaves were dried in an air-drying oven for 3 days at 45 °C [16]. The aqueous extraction of phenolic compounds was conducted with deionized distilled water (ddH_2_O) (1:20 *w*/*v*) and stirred at 45 °C for 60 min, then centrifuged at 3000× *g* for 15 min (Centrifuge model K241R, Centurion Scientific Ltd., Chichester, UK) and filtered with filter papers (Wattman#1). Supernatant was collected, filtered and lyophilized (Freeze dryer FDF 0350; Seoul, Republic of Korea) [17].

### 2.3. Total Phenolic and Flavonoids Content

Folin–Ciocalteu assay was used to estimate the total phenolic content, as stated in [18]. The Folin–Ciocalteu reagent and 0.5 mL ddH_2_O were added to 500 μL of dissolved OLE. Then, 1.25 mL of 7% Na_2_CO_3_ was added after the mixture had been shaken and left to stand for 6 min. The volume was then brought up to 3 mL with ddH_2_O, and the mixture was left in a dark place for another 30 min. A 650 nm measurement was made of the mixture’s absorbance. Different gallic acid concentrations (0–1000 µg/mL) were used as a standard, and a standard curve was produced. Total phenolic content was expressed for µg GAE/mg of dry sample. 

Total flavonoid content was estimated following the method of Dewanto et al. [18] by mixing OLE (250 μL) with 75 μL NaNO_2_ (5%). After standing for 6 min, 150 μL of 10% AlCl_3_ and 500 μL of 1 M NaOH were added to the mixture. Using deionized distilled water (ddH_2_O), the final volume was adjusted to 2.5 mL. The absorption of the mixture was noted at 510 nm using Shimadzu UV-1800 UV/Visible Scanning Spectrophotometer (115 VAC, Cole-Parmer, East Bunker Ct, Vernon Hills, IL, USA). In comparison with the calibration curve using catechol as a standard, the total flavonoid content was estimated as µg CE/g extract.

### 2.4. Antioxidant Activity

#### 2.4.1. DPPH Assay

DPPH free radicals scavenging activity was determined following the method of Shimada et al. [19]. Serial concentrations of OLE (5–200 g/mL) were reconstituted. Then, 1 mL of each concentration was mixed with 1 mL of 0.078 mM DPPH. After shaking, the mixture was allowed to react in a dark area for 30 min. The mixture’s absorbance was measured at 517 nm. Ascorbic acid was used as a standard antioxidant. The DPPH radical inhibition was calculated according to the following equation:

*Inhibition* (%) = [(*Abs_control_* − *Abs_sample_*)]/(*Abs_control_*)] × 100

where *A_control_*: absorbance of the control solution; *A_sample_*: absorbance of the extract.

A linear regression analysis was used to calculate the IC_50_ (minimum inhibitory concentration) value (µg/mL). The lower the IC_50_, the higher the antioxidant power.

#### 2.4.2. Reducing Power

The method of Ferreira et al. [20] was followed to assess the antioxidant activity of the olive leaf extract. A total of 2.5 mL of phosphate buffer (0.2 M, pH 6.6) and 2.5 mL of 1% potassium ferricyanide were added to 1 mL of OLE. The mixture was incubated in a water bath for 20 min at 50 °C. After cooling, 2.5 mL of 10% trichloroacetic acid was added and the mixture was centrifuged at 3000× *g* for 10 min. Then, 2.5 mL of freshly made 0.1% ferric chloride solution was added to the solution and then an equivalent volume of ddH_2_O was added. The mixture was then left to stand for 10 min at room temperature. Similar steps were followed to prepare the control. The absorbance was noted at 700 nm. A calibration curve was constructed using ascorbic acid as a standard. EC_50_ was calculated and expressed as (μg/mL). The lower the EC_50_, the higher the antioxidant power. 

#### 2.4.3. Nitric Oxide Scavenging Activity

Nitric oxide radical inhibition was estimated using Griess Illosvory reaction (5% 1-napthylamine) following the method of Chakraborthy [21]. The reaction mixture (3 mL) (containing 2 mL of 10 mM sodium nitroprusside, 0.5 mL saline phosphate buffer and 0.5 mL of OLE extract) was incubated at 25 °C for 150 min. After incubation, 0.5 mL of the reaction mixture was mixed with 1 mL sulfanilic acid reagent (0.33% in 20% glacial acetic acid) and allowed to stand for 5 min for the completion of the reaction of diazotization. After this, a further 1 mL of the 5% 1-napthylamine was added, mixed and allowed to stand for 30 min at 25 °C. The absorbance of the mixture was measured at 546 nm and nitric oxide radical inhibition was calculated using the following formula:

$$NO\;scavenged\;(\%\;inhibition)=\frac{\left(Abs_{Control}-Abs_{Sample}\right)}{Abs_{Control}}\times 100$$

### 2.5. HPLC Analysis of Phenolic Compounds

High performance liquid chromatography analysis was performed for the analytical qualification and quantification of phenolic compounds in each olive leaf extract according to the IOOC method [22]. The extracts (10 mg) were dissolved in 80% methanol (1 mL) and filtered with 0.45 μm filters before analysis using HPLC (Agilent1260 infinite HPLC Series, Agilent, CA, USA). The separation of phenolic compounds was performed on a C18 column (aKinetex^®^ 5l Jm EVO C18: 25 mm × 4.6 mm, Phenomenex, Torrance, CA, USA) at a temperature of 40 °C. The elution was carried out in gradient mode using a binary solvent mixture composed of water acidified with 0.2% phosphoric acid (solvent A) and methanol/acetonitrile 50/50 (solvent B). A linear gradient was run from 96% (A) and 4% (B) to 50% (A) and 50% (B) during 40 min; it changed to 40% (A) and 60% (B) for 5 min; and during 15 min it changed to 0% (A) and 100% (B), after re-equilibration for 12 min to the initial composition. The mobile phase flow rate was 1 mL/min and the injection volume of each sample was 20 μL. All phenolic compounds were identified by comparing their retention times with those of standards (Gallic acid, Catechol, *p*-Hydroxy benzoic acid, Caffeine, Vanillic acid, Caffeic acid, Syringic acid, Oleuropein, Vanillin, *p*-Coumaric Acid, Ferulic acid, Rutin, Ellagic acid, Benzoic acid, o-Coumaric acid, Salicylic acid and Cinnamic acid). The concentration of phenolic compounds was given as mg/g sample.

### 2.6. In Vitro α-Glucosidase Inhibitory Activity

The method of Wresdiyati, et al. [23] was followed to determine the inhibitory power of α-glucosidase activity. A 0.1 M quantity of phosphate buffer (pH 7.4) and 10 µL aliquot of OLE (1 mg/mL) were added to 100 µL of α-glucosidase and the mixture was incubated in a water bath at 30 °C for 5 min. Then, 500 µL of PNPG (5 mM chromogenic α-glucuronidase substrate) was added and the mixture left to stand for 15 min at room temperature. The stop reaction (2 µL of 1 M Na_2_CO_3_) was added and the absorbance was read at 400 nm after identical preparations for the control and blank. The amount of *p*-nitrophenol nanomoles emitted in a minute was used to determine the enzyme activity unit.

% *Inhibition* = {1 − (*AB test sample*/*AB control*)} × 100

### 2.7. Hemolytic Activity Assay

Blood was drawn from healthy rabbits, and then, after centrifuging at 5000 rpm for 10 min, the obtained erythrocytes were washed three times with phosphate-buffered saline (PBS). Then, the final cell suspension (which had a 5% hematocrit) was kept at 4 °C for further use. Hemolysis of the red blood cells (RBCs) is due to the oxidative stress caused by free radicals. These free radicals could be neutralized by antioxidant compounds. The reduction of the hemolysis by OLE was assessed using the method of Afsar et al. [24]. A red blood cell suspension (1% hematocrit) was incubated with different concentrations of the extract (50–500 µg/mL) over 30 min at 37 °C. After centrifugation, the resulting erythrocytes were next incubated for 6 h with AAPH, and then the supernatant obtained after centrifugation was used to determine the level of hemolysis by measuring the absorbance of liberated hemoglobin from the cell at 540 nm in the tested extract and control sample (treated cells with AAPH only). As a reference antioxidant material, ascorbic acid was used. Lastly, the percentage of antihemolysis (% inhibition of hemolysis) was calculated using the following equation:

% of hemolysis inhibition = [Abs_cont_ − Abs_sample_/Abs_cont_] × 100

where Abs_cont_ is the control’s absorbance (treated cells just with AAPH, no extract) and A_sample_ is the tested extract’s absorbance.

### 2.8. Animal Models and Experimental Design

For the in vivo experiment, adult male rats (8 weeks old, 200–220 g weight) were purchased from the animal house of the Institute of Graduate Studies and Research, Alexandria University, Egypt. Animals had unlimited access to food and drink while they were being experimented on. The properties of the cages that held the animals were: enclosure height 30 cm, enclosure length 50 cm and enclosure area 1500 cm^2^. The Institutional Animal Care and Use Committee (AlEXU-IACUC) at Alexandria University is part of the International Council for Laboratory Animal Science (ICLAS), and gave its approval for all the experimental methods to be carried out (permission number: AU:141901160205 on 16 January 2019). The experiment was continued for one month after the one-week acclimation period. Streptozotocin (STZ) was injected into rats to cause diabetes at a dose of 40 mg/Kg body weight. Due to its superior phenolic content and antioxidant activity, olive leaf extracts (OLE) (200 mg extract/Kg body weight) were used for the in vivo experiment. Metformin was used as a standard medication at the dosage of 200 mg/Kg body weight. The rats were grouped in five groups of six rats each. All rat groups were given water and commercial pellet meals with a crude protein content of 23%, crude fat content of 3%, crude fiber content of 7%, and ash content of 8%. The experimental design was as follows:

1. Group A: designated as a Negative Control (NC), rats received 1 mL of saline orally and were fed pellets.
2. Group B: Positive Control (PC), diabetic rats with diabetes that had been induced with STZ received 1 mL of saline orally and were fed pellets.
3. Group C: STZ (diabetic rats) received a metformin (200 mg) orally once a day.
4. Group D: STZ (diabetic rats) received OLE (200 mg) orally once a day.
5. Group E: STZ (diabetic rats) received a mix of OLE (100 mg) and metformin (100 mg) once a day.

The experiment was continued for four weeks and the rats were slaughtered at the end of the experiment under light Anhalt anesthesia in accordance with the ethical committee’s rules [25]. The blood samples were drawn into heparinized tubes and centrifuged at 3000× *g* for 10 min and then the samples were transferred in an ice box to the Mabaret El-Asafra Clinical Laboratories in Alex, Egypt for biochemical analysis. Livers, kidneys, and pancreas were collected and subsequently fixed in 10% neutral buffered formalin solution for histopathological analysis.

### 2.9. Biochemical Analysis

All biochemical analyses—blood glucose level, HbA1c, liver function parameters, kidney function parameters and lipid profile—were conducted in an accredited laboratory (Mabaret El-Asafra Clinical Laboratories in Alexandria, Egypt) according to the official clinical analysis of Rowbottom [26].

### 2.10. Histopathology

Liver, kidney and pancreas samples from all groups were subsequently fixed in 10% neutral buffered formalin solution. After 24 h, the material was dehydrated in escalating ethanol, cleaned in xylene, and finally embedded in paraffin wax. A light microscope (ZEISS Axioscope 5 digital pathology microscope, Carlsbad, CA, USA) was used to evaluate the histopathology of tissue sections (3–5 microns thick), which were cut and stained with hematoxylin and eosin (H&E) according to the method of Bancroft and Stevens [27].

### 2.11. Statistical Analysis

SPPS software version 16, New York, NY, USA, was used for statistical analysis. Duncan’s test was used to find significant differences between all of the studied parameters using a one-way analysis of variance with a *p*-value ≤ 0.05. All experiments were conducted in triplicate.

## 3. Results and Discussion

### 3.1. Total Phenolic and Flavonoids Content

The data presented in Table 1 show the total phenolic and flavonoids content of the extract of the three Egyptian genotypes of olive leaves (picual, tofahi and shamlali). The findings reported here shed new light on the significant phenolic and flavonoids content of the three studied genotypes. The highest level of both phenolic and flavonoids content was found in the picual leaves’ extract (115.00 ± 4.00 µg GAE/g and 559.00 ± 1.00 µg CE/g), followed by tofahi (75.34 ± 0.21 µg GAE/g and 49.38 ± 0.16 µg CE/g) and shamlali (60.00 ± 0.50 µg GAE/g and 42.00 ± 0.60 µg CE/g), respectively (*p* < 0.05). The findings of the current investigation agree with several previous studies [28,29,30]. The differences in the phenolics and flavonoids content among three genotypes might be due to genetic variations which might affect the biosynthesis process and the accumulation of phenolic compounds in the plant [31]. Numerous health benefits have been linked to a phenolic-rich diet, due to their ability to reduce oxidative stress, scavenge free radicals and chelate metal ions, and due to their ability to modulate intracellular signaling pathways [32]. These health benefits of phytochemicals make the olive leaf extracts effective and beneficial for several nutritional and pharmaceutical applications.

### 3.2. Antioxidant Activity

#### 3.2.1. DPPH Assay

Table 2 shows the DPPH scavenging activities of the three genotypes of OLE: picual, tofahi and shemlali. All three types of extracts showed a considerable scavenging activity, with IC_50_ 48.14 ± 0.15, 56.00 ± 0.10 and 56.00 ± 0.13 μg/mL for picual, tofahi and shemlali, respectively. There were no significant differences between tofahi and shemlali (*p* > 0.05), while picual extract showed the lowest IC_50_ value and the highest DPPH scavenging activity (*p* ≤ 0.05). On the other hand, the IC_50_ of ascorbic acid as an antioxidant standard was still lower than the three OLEs (11.16 ± 2.00 µg/mL). The reasonable and effective antioxidant power of OLEs might be due to the diversity and nature of phenolic compounds and flavonoids which have a good ability to neutralize the free radicals and prevent the oxidation process. These outcomes are consistent with earlier conclusions that emphasized the strong DPPH radical scavenging ability of olive leaf extracts, even in low concentrations [33].

#### 3.2.2. Reducing Power

Table 2 shows the reducing power of the three olive leaf extracts (picual, tofahi, shamlali). The reducing power showed no significant differences between the three extracts (EC_50_: 53.11, 51.21 and 55.16 μg/mL, respectively) compared to ascorbic acid as a standard (EC_50_ = 15.57 μg/mL), which showed the lowest EC_50_. It is noticeable that the antioxidant activity using DPPH and reducing power showed very close results. The remarkable reducing power of the OLEs indicates the effectiveness of the phenolic compounds and flavonoids, which might be beneficial in the treatment and/or prevention of several human diseases and which might have health promoting properties. This result emphasized previous work, which reported the high antioxidant activity and oxidative stability of olive leaf extracts [34,35,36]. Several in vitro and in vivo studies have reported phenolic compounds as showing anti-inflammatory, anti-microbial, anti-diabetic and anti-tumoral properties.

#### 3.2.3. Nitric Oxide Scavenging Activity

One of the inflammation implications in human cells is the release of lysosomal enzymes, which causes different kinds of disorders and pathological conditions [37,38]. The extracts of three Egyptian genotypes of olive leaves (picual, tofahi and shemlali) showed high nitric oxide scavenging activities. Picual leaf extract showed an IC_50_ of 19.03 ± 0.13 μg/mL, the tofahi leaf extract showed an IC_50_ of 20.00 ± 0.30 μg/mL and shemlali showed an IC_50_ of 20.32 ± 0.11 μg/mL (Table 2). All three OLEs showed an IC_50_ lower than quercetin (3.00 ± 0.01 μg/mL) as a standard (*p* ≤ 0.05). The high frustration ability of OLE on NO^-^ formation is due to the high antioxidant power of phenolic compounds and, in turn, may have an effective role in preventing the implications of oxidative stress of excessive NO^-^ in the living cells. Additionally, the scavenging activity of OLE may be able to stop the potentially hazardous “NO” radical-initiated oxidation chain reaction [39]. Martínez-Navarro et al., 2023 [40], revealed that olive leaf extract (OLE) is an effective antioxidant in biological systems, preventing oxidative stress based on its scavenging activity against RNS, with an IC_50_ of 48.4 ± 6.8 mg/mL. The nitric oxide scavenging activity showed a lower IC_50_ than DPPH and reducing power, which suggests that the NO^-^ radicals are more sensitive against the phytochemical molecules of OLE.

### 3.3. Identification of Phenolic Compounds by HPLC

The method is based on the direct extraction of the phenolic compounds from olive leaf extract by means of a methanol solution and subsequent quantification using HPLC with the aid of a UV detector at 280 nm. The standard contained a mix of 17 different phenolic compounds (Gallic acid, Catechol, *p*-Hydroxy benzoic acid, Caffeine, Vanillic acid, Caffeic acid, Syringic acid, Oleuropein, Vanillin, *p*-Coumaric Acid, Ferulic acid, Rutin, Ellagic acid, Benzoic acid, o-Coumaric acid, Salicylic acid and Cinnamic acid). All phenolic compounds were identified by comparing their retention times with those of standards. The HPLC analysis of the three OLEs showed a significant amount of different phenolic compounds with different concentrations (Table 3). Oleuropein, rutin, benzoic acid, salicylic acid and *p*-hydroxy benzoic acid were the predominant photochemical compounds in each of the three OLEs, while caffeic acid, vanillic acid, vanillin, *p*-coumaric acid, ellagic acid and *o*-coumaric acid showed moderate concentrations. On the other hand, gallic acid, catechol, caffeine, syringic acid, ferulic acid and cinnamic acid showed the lowest concentration levels of phenolic compounds (Table 3). Oleuropein was the most abundant phenolic compound in all three extracts, with concentrations of 3515.00 ± 8.00, 3617.00 ± 16.00 and 3849.00 ± 8.00 µg/g in picual, tofahi and shamlali, respectively. The oleuropein might play a key role in the antioxidant activity of the OLEs. The antioxidant potential of oleuropein may be due to its ability to chelate metals ions, such as Cu and Fe, that catalyze free radical generation reactions, as well as the ability to inhibit many inflammatory enzymes such as lipoxygenases without affecting the cyclo-oxygenase pathway [41]. Orak et al., 2019 [42], studied the phenolic compounds in 12 olive leaf genotypes and showed that oleuropein was the primary component for all genotypes. These findings were in line with the findings of Martínez-Navarro et al. [40], who revealed that the oleuropein showed the highest value in the ‘Picual’ genotype compared to other genotypes. Zhang et al. [43] identified thirty-two phytochemical compounds in olive leaf extracts using high-performance liquid chromatography–electrospray ionization–tandem mass spectrometry, including seventeen flavonoids, five iridoids, two hydroxycinnamic acids, six triterpenic acids, one simple phenol and one coumarin. Olive leaves were found to be excellent sources of flavonoids, iridoids and triterpenic acids, and considerable variations in phytochemical content were detected among the different cultivars.

### 3.4. α-Glucose Oxidase Inhibitory Properties

Among the most common types of blood-sugar-lowering drugs are α-glucosidase inhibitors (AGI). α-glucosidase enzyme exists at the brush border of the small intestine and is responsible for carbohydrate breakdown and monosaccharide absorption. AGI slows down the absorption of ingested carbohydrates, reducing glucose and insulin spikes after meals [44]. The evaluation of the α-glucosidase inhibitory effect of olive leaf extracts in the present study may contribute to the understanding of their mechanism of action in reducing blood sugar and developing alternative natural and safe antidiabetic food supplements. In the current investigation, all tested OLEs showed a strong α-glucosidase inhibitory activity (*p* ≥ 0.05). The inhibition of α-glucosidase was increased with an increasing concentration of OLE (25–100 μg/mL). The minimum inhibitory concentrations (IC_50_) of OLE that showed a 50% inhibition of α-glucosidase activity were 14.14 ± 0.41, 14.35 ± 0.22 and 14.61 ± 0.28 μg/mL for picual, tofahi and shemlali extract, respectively (*p* ≤ 0.05) (Table 4). AlShaal et al., 2020 [45], reported that the olive leaf extracts showed an 81.34% inhibition of α-glucosidase at a concentration of 3.85 mg/mL with IC_50_ = 0.34 ± 0.12 mg/mL. The hydroxytyrosol and oleuropein of olive leaves showed a strong α-glucosidase inhibitory effect with IC50 values of 600 mM and 400 mM, respectively, which emphasized their potential effect as α-glucosidase inhibitors for the management of postprandial hyperglycemia [46]. Caffeic acid, curcumin, cyanidin, daidzein, epicatechin, eridyctiol, ferulic acid, hesperetin, narenginin, pinoresinol, quercetin, resveratrol and syringic acid can significantly inhibit the α-glucosidase enzyme [47]. The α-glucosidase inhibitory activity of OLEs in this study may be due to their phenolic compounds, which are able to bind with the protein part of the enzyme (apoenzyme), causing the hindrance of its function. Numerous in vitro investigations have demonstrated that carbohydrate hydrolytic enzyme inhibition might be caused by plant polyphenols that bind to proteins [47,48,49]. This finding emphasized the crucial role of the phenolic compounds in OLEs in delaying the hydrolysis and absorption of monosaccharides by the inhibition of α-glucosidase activity, consequently lowering the blood glucose level.

### 3.5. Anti-Hemolytic Activity

The hemolytic activity assay was performed in a 1% erythrocyte suspension. The degree of hemolysis of the HEBCs membrane in the presence of OLEs is presented in Table 5. The obtained results showed that the degree of hemolysis decreased when increasing the dose of the OLE. The lowest degree of hemolysis (the highest stability of the RBCs’ membrane) (3.40 ± 0.17, 3.80 ± 0.11 and 3.80 ± 0.13%) was found with 200 μg/mL (*p* ≤ 0.05). On the other hand, the lowest RBCs stability was noted with the lowest dose of OLE (50 μg/mL). The protective effect on the RBCs’ membrane by OLEs is dose dependent. The oxidative status of cells is determined by the balance between pro-oxidants and antioxidants. Pro-oxidants, referred to as reactive oxygen species (ROS), are classified into radicals and nonradicals. The radicals are highly reactive due to their tendency to accept or donate an electron and attain stability. When cells experience oxidative stress, ROS, which are generated in excess, may oxidize proteins, lipids and DNA, leading to cell death and organ damage. Oxidative stress is believed to aggravate the symptoms of many diseases, including hemolytic anemias [50]. The protective effect of OLE is due to its polyphenols, which can neutralize ROS and other free radicals by donating electrons and protecting the cell against their attack. OLE is an important source of natural antioxidants; it has effective antioxidant activity against different reactive species, and protects human erythrocytes against oxidative damage [51]. Natural phytochemicals can reduce hemolysis and could be used to prevent and treat hemolytic anemias [52].

### 3.6. Biochemical Investigation 

#### 3.6.1. Glucose and HbA1c Levels in Diabetic Rats

The total glucose test and HbA1c test are common and useful parameters for controlling diabetes in patients. Metformin is a diabetic drug which helps to control the amount of glucose in your blood by decreasing the amount of glucose absorbed from food and decreasing the amount of glucose made by the liver. The effects of OLE, metformin and their combination on glucose and HbA1c levels are shown in (Table 6). The levels of glucose and HbA1c in streptozotocin-induced rats (PC) recorded a significant increase compared to the negative control (NC) (340.00 ± 6.82 mg/dL and 13.80 ± 0.20%) (*p* < 0.05). In contrast, the administration of OLE alone and the combination of OLE and metformin recovered the level of glucose and HbA1c to the normal level even more effectively than metformin alone. The group treated with metformin alone showed a reduction in the level of total blood glucose to 154.00 ± 4.10 mg/dL and HbA1c to 4.80 ± 0.10%. In the diabetic group treated with OLE, the level of blood glucose significantly decreased to 174.33 ± 7.14 mg/dL and HbA1c to 4.80 ± 0.10%. On the other hand, in the group of diabetic rats treated with the combination of metformin and OLE (1:1), the level of blood glucose significantly decreased to 121.67 ± 5.49 mg/dL and HbA1c to 4.70 ± 0.10%. From the obtained results, it can be noted that OLE showed an ability to recover the level of sugar in diabetic rats that was close to the normal level at the dose of 200 mg once a day. The effect of OLE was close to the effect of metformin as a standard medication, but the superiority of OLE was that it is a natural product and has no side effects. Notably, the combination of metformin and OLE demonstrated a significant decrease in total blood glucose level and HbA1c, better that both metformin and OLE alone (121.67 ± 5.49 and 4.70 ± 0.10) (*p* < 0.05). This emphasizes that the OLE might give the best results if it is used as an adjuvant agent with standard antidiabetic drugs. The use of olive leaf extract (OLE) may regulate the level of blood glucose in diabetic rats induced by STZ by enhancing the regeneration of β-cells of the pancreas [53]. Olive leaf extract is associated with improved glucose homeostasis in humans through the reduction of starch digestion and absorption, and may represent an effective adjunct therapy that normalizes glucose homeostasis in individuals with diabetes [15]. Another study suggested that OLE exerted antihyperglycemic effects via AS160 inhibition, and it could be used as an alternative to metformin treatment [54]. The use of OLE alone or in combination with metformin can control the blood glucose levels in diabetic patients.

#### 3.6.2. Lipid Profile of Diabetic Rats

Patients with diabetes mellitus usually suffer from lipid abnormalities, often called “diabetic dyslipidemia”, which are typically characterized by high total cholesterol (T-Chol), high triglycerides (Tg), low high-density lipoprotein (HDL-C) and high levels of low-density lipoprotein (LDL). The impact of OLE on the lipid profile of induced diabetic rats is presented in Table 7. The total cholesterol level in streptozotocin-induced diabetic rats (PC) showed a considerable elevation in total cholesterol (174.00 ± 4.58 mg/dL) compared to the negative control group (60.00 ± 4.50 mg/dL) (*p* ≤ 0.05) but did not exceed the normal level (≤200 mg/dL). The same trend was noted with the level of triglycerides (152.00 ± 1.73 mg/dL) and LDL (120.15 ± 0.33 mg/dL), and HDL, which was reduced from 54.00 ± 3.21 mg/dL in the NC group to 40.00 ± 3.06 mg/dL in the PC group. The administration of OLE led to a significant reduction in the levels of total cholesterol (52.00 ± 1.73 mg/dL), triglycerides (118.33 ± 2.89 mg/dL) and LDL (60.36 ± 0.33 mg/dL), bringing them closer to the levels of the NC group. It is worth noting that the OLE recovered the lipid profile better that metformin (Table 7). The combination of metformin and OLE resulted in a significant decrease in total cholesterol to 49.33 ± 1.45 mg/dL, in the triglyceride to 109.00 ± 3.60 and in LDL to 51.46 ± 0.33 mg/dL, respectively (*p* ≤ 0.05). On the other hand, there was no significant differences in the level of HDL between treated groups and control groups (*p* ≥ 0.05). Interestingly, the lipid profile parameters did not register very high values, due to the composition of the diet not being high fat. Research shows that olive leaf extract helps to prevent LDL from building up in the arteries. This effect helps to increase blood flow and lower blood pressure, reducing your risk of heart disease [55]. The study of Fki et al. revealed that the administration of 10 mg/kg of body weight of olive mill wastewater extract significantly lowered the total cholesterol serum level and LDL-C while increasing the serum levels of high-density lipoprotein cholesterol (HDL-C) [56]. The administration of OLE containing 200 mg oleuropein for 8 weeks in patients with stage-1 hypertension resulted in a reduction in total cholesterol, LDL-cholesterol, and triglyceride levels [57]. The hypocholesterolemic effect of olive leaf extracts might be due to their abilities to lower serum, total cholesterol and LDL-C levels, as well as slowing the lipid peroxidation process and enhancing antioxidant enzyme activity.

#### 3.6.3. The Liver Functions of Induced Diabetic Rats

**Figure 1:**
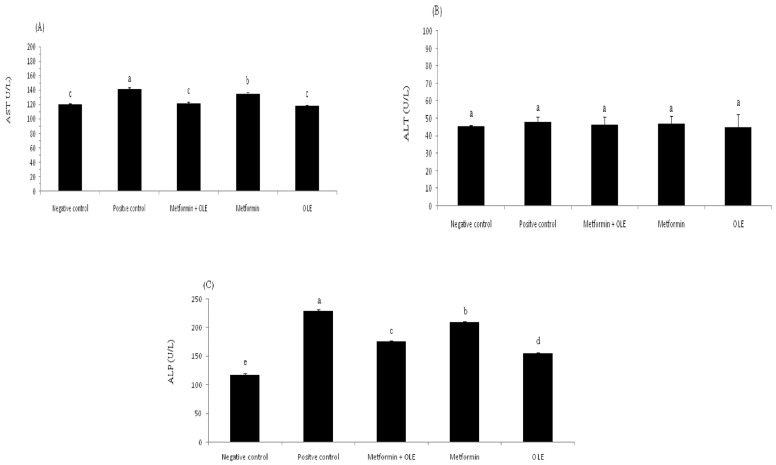
Liver enzyme analysis of diabetic rats. where each reported value is the mean ± SD of replicates with significant difference if *p* ≤ 0.05. OLE: olive leaf extract, NC: negative control; and PC: positive control. (**A**) Aspartate transaminase (AST) level; (**B**) Alanine transaminase (ALT) level; and (**C**) Alkaline phosphatase (ALP) level.

Diabetes raises the risk of nonalcoholic fatty liver disease. People living with type 2 diabetes are usually under the risk of nonalcoholic fatty liver disease. The analysis of liver enzymes (AST, ALT and ALP) for induced diabetic rats during the experiment is presented in Figure 1. The analysis of liver enzymes showed a considerable elevation in the AST level in induced diabetic rats (PC group), up to 141.67 ± 1.53 U/L compared to the NC group 120.33 ± 1.15 U/L (*p* ≤ 0.05), which reflects the direct relationship between diabetes mellitus and liver functions (Figure 1A). In contrast, the administration of OLE or the combination of OLE + metformin showed significant repair to the AST levels (118.00 ± 1.73 and 121.33 ± 2.31 U/L, respectively), bringing them close to the level of the NC group. There were no significant differences between OLE, metformin and the mixture of OLE and metformin (*p* ≥ 0.05). The same trend was noted with the level of ALP and ALT, as well, where the administration of OLE and OLE and metformin corrected the level of ALP and ALT to bring it close to the NC group (Figure 1B,C). It is worth mentioning here that the OLE showed a superiority compared to metformin in repairing the liver functions, which might be due to the antioxidant power of its phenolic compounds, which plays a crucial role in removing the adverse effects of oxidative stress. Previous research has emphasized OLE’s ability to repair liver enzymes, which may be related to its antioxidant activity, which helps to prevent diabetes complications associated with oxidative stress [58].

#### 3.6.4. Kidney Function in Diabetic Rats

**Figure 2:**
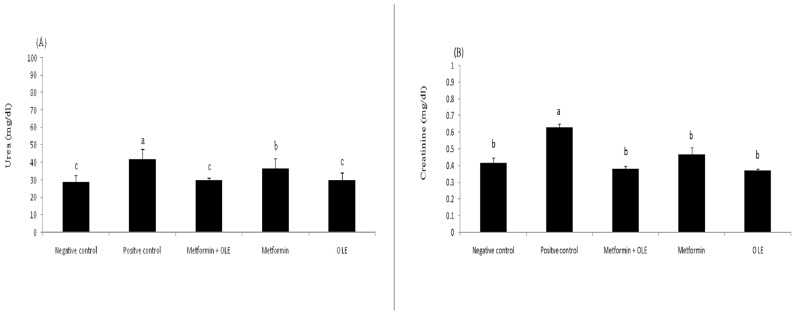
Kidneys functions of diabetic rats, where each reported value is the mean ± SD of replicates with significant difference if *p* ≤ 0.05. (**A**) shows the level of urea in NC, PC and treated groups; (**B**) shows the level of creatinine in in NC, PC and treated groups.

Dietary protein converts to urea and creatinine during the metabolism process and is excreted exclusively through the kidney. In diabetic patients, high blood glucose can damage the blood vessels of the kidneys, causing kidney failure and, consequently, causing the accumulation of urea and creatinine in the blood. Therefore, the determination of the level of blood urea and creatinine is an indicator for the status of the kidneys. The results in Figure 2 show the analysis of urea and creatinine in the kidneys of induced diabetic rats. The levels of creatinine and urea were significantly increased in the STZ-induced diabetic rats (PC), up to 0.63 ± 0.02 mg/dL and 41.67 ± 5.94 mg/dL compared to the negative control (NC) (0.42 ± 0.03 mg/dL and 29.00 ± 3.46, respectively (*p* ≤ 0.05). Using OLE, metformin and their combination at the dose level of 200 mg resulted in a significant remediation in the creatinine (0.37 ± 0.01, 0.47 ± 0.04, 0.38 ± 0.02 mg/dL) and urea (0.37 ± 0.01, 0.47 ± 0.04, 0.38 ± 0.02 mg/dL), respectively, bringing them close to the NC group with no significant differences between them (*p* ≥ 0.05) (Figure 2A,B). Experimentally, OLE extract has a superior pronounced effect in repairing the creatinine and urea levels in the kidneys, which might return antioxidant properties, which removes oxidative stress, causing the survival of blood vessels of the kidneys in a healthy state. Previous work revealed that OLE has a real ability to correct hepatic or renal functions, and might be considered a promising therapeutic agent [59].

### 3.7. Histopathologic Examination

**Figure 3:**
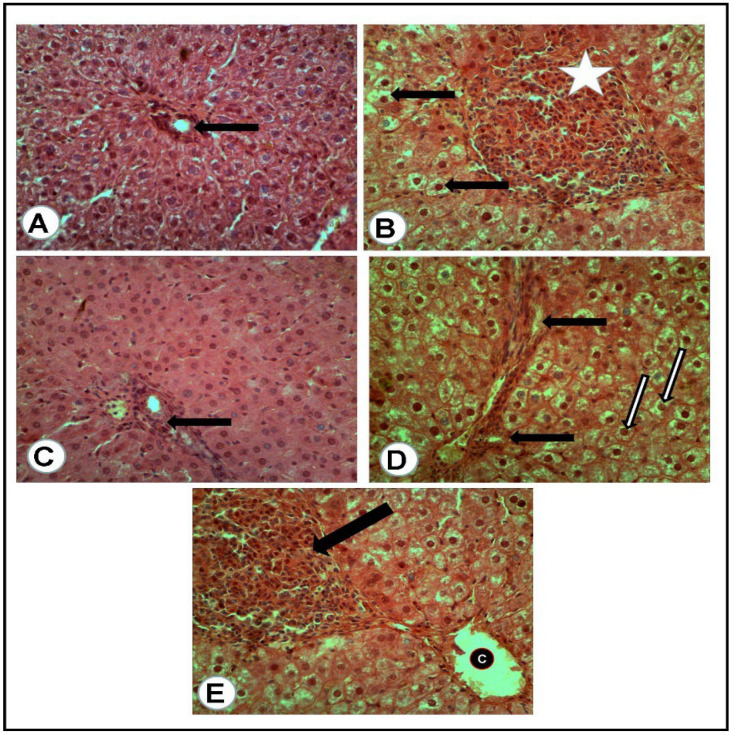
Liver tissues of rats of all groups (H&E, ×400): (**A**) negative control group (NC): normal portal area (arrow) with surrounding hepatic cells. (**B**) Positive control (PC) group induced with STZ: portal area severely loaded with mononuclear cell aggregation and small hyperplastic bile ducts (white asterisk) while most of all the hepatic cells suffered vacuolar and hydropic degeneration (arrows). (**C**) Group treated with the combination of (metformin and OLE), with the portal area containing some actively infiltrated mononuclear cells. (**D**) Group treated with metformin alone: numerous newly formed bile ducts (black arrows) in addition to vacuolar and hydropic degeneration of the hepatic cells (white arrows). (**E**) Group treated with (OLE) alone: clear dilated central vein (C) with nearly normal surrounding hepatocytes and presence of large focus of mononuclear cell aggregation (Black arrow).

**Figure 4:**
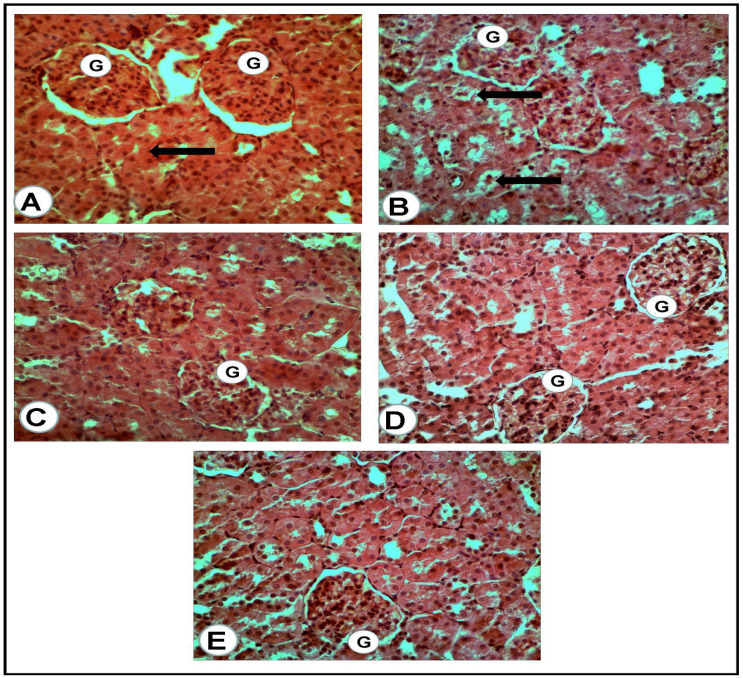
Renal cortex of the rats of all groups (H&E, ×400): (**A**) negative control group: normal glomeruli (G) and proximal convoluted tubules (arrow). (**B**) Positive control for STZ treated group: the glomeruli (G) appeared hypercellular, while most of the present proximal convoluted tubules appeared dilated and contained cast formations (black arrows). (**C**) Treated group with mix (metformin and extract): the glomeruli (G) appeared hypercellular, while the proximal convoluted tubules were nearly normal. (**D**) Treated group with (metformin and STZ): the glomeruli (G) appeared swollen and hypercellular, while a mild degeneration of the tubular uroepithelium was seen. (**E**) Treated group with (extract and STZ): the glomeruli (G) appeared nearly normal, less cellular with surrounding normal uroepithelium of the proximal renal tubules.

**Figure 5:**
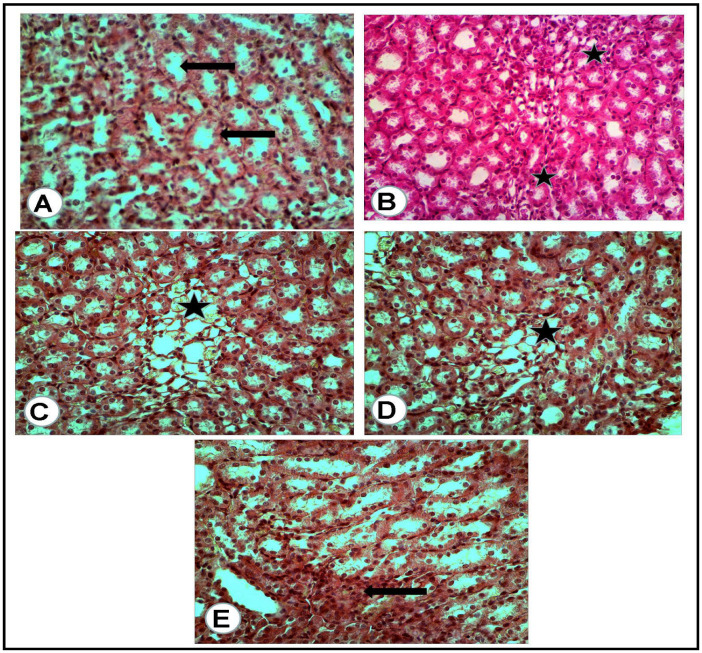
Renal medulla of the rats of all groups (H&E, ×400): (**A**) negative control group: cross section of normal medullary tubules (arrows) with a normal uroepithelium and clear lumina. (**B**) Positive control for STZ treated group: excess mononuclear cell infiltrations (asterisks) in between a degenerated medullary tubule. (**C**) Treated group with mix (metformin and extract): excess of thin wall, dilated tubules (asterisk) with flattened uroepithelium. (**D**) Treated group with (metformin and STZ): few dilated tubules (asterisk) with flattened uroepithelium. (**E**) Treated group with (extract and STZ): some of the medullary tubules appeared with high and voluminous uroepithelium (arrow).

**Figure 6:**
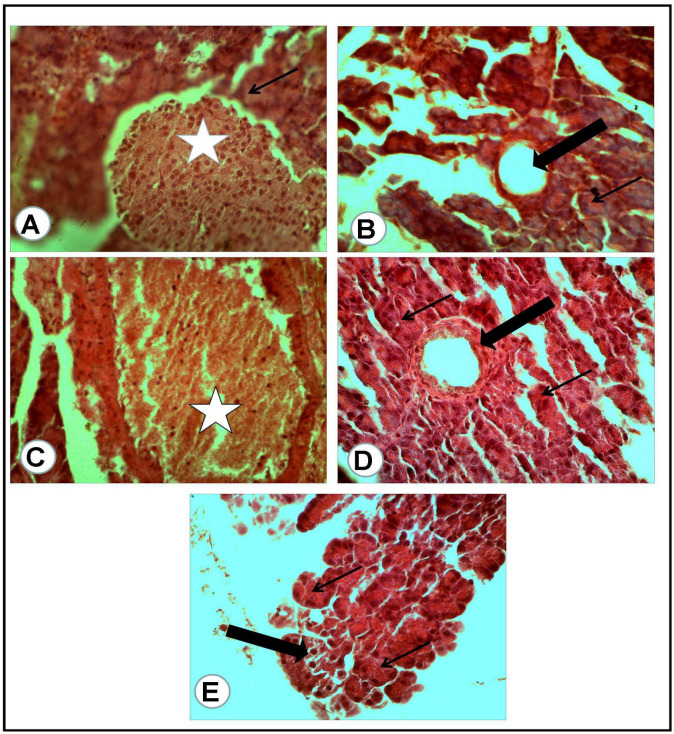
Pancreatic tissue of rats of all groups (H&E, ×400): (**A**) negative control group: normal cells of some islets of Langerhans (white asterisk) with a normal surrounding pancreatic acinus (arrow). (**B**) Positive control for STZ treated group: completely degenerated and necrotic islet of Langerhans replaced by cystic structure (thick arrow), with surrounding nearly normal pancreatic acini (thin arrow). (**C**) Treated group with mix (metformin and extract): the islet of Langerhans appeared active and large (white asterisk) and surrounded by congestion. (**D**) Treated group with (metformin and STZ): partially degenerated and necrotic islet of Langerhans (thick arrow) with nearly normal surrounding pancreatic acini (thin arrows). (**E**) Treated group with (extract and STZ): the degenerated islet replaced by a few large and mononuclear cells (thick arrow) in between active secretory pancreatic acini (thin arrows).

The microscopic examination of the organs of healthy rats in the negative control group, in the present work, revealed the presence of normal histologic criteria in the liver, kidneys and pancreatic tissues (Figure 3A, Figure 4A, Figure 5A and Figure 6A). Similarly, these organs in rats treated with the combination of metformin and OLE exhibited nearly normal and active microscopic criteria (Figure 3C, Figure 4C, Figure 5C and Figure 6C). Some defensive mononuclear cells were seen infiltrating from the portal areas in the livers, or the periglomerular cells in the kidneys, while the islet of Langerhans, in the pancreas, appeared active and large. The histopathologic examination of the organs of diabetic rats (PC) revealed the presence of obvious changes in liver tissues which showed severe portal mononuclear cell aggregation and biliary hyperplasia, in addition to hepatocytic vacuolar and hydropic degeneration (Figure 3B). Kidneys showed glomerular hypercellularity, mononuclear cell aggregation, tubular degeneration and cast formation (Figure 4B and Figure 5B) and pancreatic islets of Langerhans (cellular degeneration and necrosis) (Figure 6B). These changes were mainly and generally attributed to some degree of toxic effect of the STZ, but with regard to the pancreatic islets this was especially considered to clarify the characteristic mechanism for the diabetic effect of STZ on the endocrine cells responsible for the secretion of insulin. The microscopic examination of the organs of diabetic rats treated with metformin alone revealed a presence of some degree of changes in the liver, which showed newly formed bile ducts and hepatocytic degeneration (Figure 3D). Kidneys showed glomerular hypercellularity, dilated medullary tubules with mild degeneration and flattened uroepithelium (Figure 4D and Figure 5D), and the pancreas showed a partially degenerated and necrotic islet of Langerhans (Figure 6D). These detected changes indicate some (but not completely) ameliorating effects of metformin against the toxicity of STZ. With regard to the pancreatic islets, some partial and mild regenerative effects could be observed and may be better for relieving the diabetic effect of STZ either by repeated or longstanding treatment or by using higher doses. The microscopic examination of the organs of diabetic rats treated with olive leaf extract (OLE) alone still showed the presence of some degree of changes in the liver (fewer degenerated hepatocytes, with some large foci of mononuclear cell aggregations) (Figure 3E). Kidneys appeared nearly normal, with fewer cellular glomeruli and nearly normal uroepithelium in most of the cortical and medullary tubules (Figure 4E and Figure 5E). In the pancreas, the degenerated islet was replaced by a few large and mononuclear cells in between the active pancreatic acini (Figure 6E). These detected changes also indicated the less-complete ameliorating effect of the olive leaf extract against the toxicity of STZ. With regard to the pancreatic islets, fewer or no regenerative effects were observed. 

## 4. Conclusions

The current work aimed to evaluate the antioxidant and antidiabetic activity of olive leaves to add value to this huge by-product and explore its potential use for nutritional and pharmaceutical applications. The current investigation revealed a significant antioxidant and hypoglycemic activity due to a high content of bioactive compounds. The extracts of three genotypes of olive leaves (picual, tofahi and shemlali) showed significant phenolic and flavonoids contents, which play a key role in their antioxidant power. The picual genotype showed the best antioxidant activity via DPPH (IC_50_ = 48.14 ± 0.15 μg/mL) and nitric-oxide-scavenging activity (IC_50_ = 19.03 ± 0.13 μg/mL). Due to the highest antioxidant activity of the picual genotype, it was used in the in vivo experiment at a dose of 200 mg. OLEs exhibited a strong α-glucosidase inhibitory activity for all three OLEs with no discernible differences (*p* ≥ 0.05). OLE and the combination of OLE and metformin effectively controlled the blood glucose level, kidney and liver functions to be within normal range. A histological examination emphasized the ability of both OLE and the combination of metformin and OLE to repair the liver, kidneys and pancreatic tissues to return them to near the normal status. The main findings of the current study lead to the possibility of using olive leaf extracts alone or as an adjuvant agent with common medications for best efficiency in the treatment protocol of diabetes mellitus type II at the dose of 200 mg once a day. Deeper and more complete preclinical studies in humans should be carried out for more evidence about the efficacy of the utilization of olive leaf extracts in the treatment of diabetes mellitus type II.

## Figures and Tables

**Table 1:** Total phenolic and flavonoids content of olive leaf extracts.

Type	TPC (µg GAE/g)	TFC (µg CE/g)
Picual	115.00 ± 4.00 ^a^	59.00 ± 1.00 ^a^
Tofahi	75.34 ± 0.21 ^b^	49.38 ± 0.16 ^b^
Shemlali	60.00 ± 0.50 ^b^	42.00 ±0.60 ^c^

Each reported value is the mean ± SD of three replicates. Means in the same column followed by different letters are significantly different (*p* < 0.05).

**Table 2:** Antioxidant activity of olive leaf extracts.

OLE *	DPPH (IC_50_ µg/mL) **	Reducing Power (EC_50_ µg/mL) ***	Nitric Oxide Scavenging Activity (IC_50_ μg/mL)
Picual	48.14 ± 0.15 ^a^	54.00 ± 0.10 ^a^	19.03 ± 0.13 ^a^
Tofahi	56.00 ± 0.10 ^b^	51.39 ± 0.20 ^a^	20.00 ± 0.30 ^a^
Shemlali	56.00 ± 0.13 ^b^	55.17 ± 0.16 ^a^	20.32 ± 0.11 ^a^
Ascorbic acid	11.16 ± 2.00 ^c^	16.00 ± 1.00 ^b^	-
Quercetin	-	-	3.00 ± 0.01

Means in the same column followed by different lower-case letters are significantly different (*p* ≤ 0.05). * OLE—olive leaf extract, ** IC_50_—concentration that is efficient and achieves 50% DPPH radical scavenging activity, *** EC_50_—the concentration at which the absorbance is effectively 0.5.

**Table 3:** HPLC phenolic compound profiles of olive leaf extract (µg/g).

Phenolic Compound	P	T	S
Gallic acid	0.23 ± 0.06 ^a^	0.20 ± 0.09 ^a^	0.19 ± 0.02 ^a^
Catechol	0.21 ± 0.08 ^a^	0.21 ± 0.05 ^a^	0.20 ± 0.02 ^a^
*p*-Hydroxy benzoic acid	375.00 ± 11.00 ^b^	392.04 ± 5.00 ^b^	411.36 ± 11.42 ^a^
Vanillic acid	58.05 ± 0.45 ^a^	55.40 ± 1.04 ^b^	59.33 ± 0.89 ^a^
Syringic acid	0.10 ± 0.01 ^b^	0.11 ± 0.01 ^b^	0.23 ± 0.03 ^a^
Oleuropein	3515.00 ± 8.00 ^c^	3617.00 ± 16.00 ^b^	3849.42 ± 8.00 ^a^
Vanillin	57.43 ± 2.43 ^b^	53.32 ± 0.60 ^b^	65.21 ± 3.19 ^a^
*p*-Coumaric Acid	25.46 ± 2.07 ^a^	18.00 ± 0.40 ^b^	26.00 ± 2.00 ^a^
Ferulic acid	0.22 ± 0.03 ^a^	0.18 ± 0.02 ^a^	0.20 ± 0.13 ^a^
Rutin	2325.00 ± 8.00 ^a^	2075.00 ± 52.00 ^b^	2325.12 ± 32.00 ^a^
Ellagic acid	123.00 ± 2.00 ^a^	119.00 ± 2.00 ^a^	120.00 ± 3.00 ^a^
Benzoic acid	860.00 ± 5.00 ^c^	932.33 ± 4.00 ^b^	1010.70 ^a^ ± 12.37
o-Coumaric acid	37.15 ± 0.66 ^a^	38.16 ± 1.74 ^a^	38.35 ± 2.17 ^a^
Salicylic acid	784.00 ± 7.00 ^c^	1375.00 ± 22.00 ^a^	1165.00 ± 11.00 ^b^
Cinnamic acid	1.54 ± 0.27 ^a^	1.22 ± 0.04 ^a^	1.51 ± 0.61 ^a^

The concentrations were reported as means of replicates ± SD. Values in the same row with different letters have significant differences (*p* < 0.05).

**Table 4:** In vitro α-glucosidase inhibitory.

Concentration (μg/mL)	% Inhibition		
	P *	T *	S *
25	88.39 ± 0.28 ^a^	87.51 ± 0.37 ^b^	85.54 ± 0.44 ^c^
50	91.51 ± 0.35 ^a^	90.55 ± 0.28 ^b^	89.59 ± 0.39 ^c^
75	92.61 ± 0.36 ^a^	91.52 ± 0.32 ^b^	91.49 ± 0.36 ^b^
100	92.59 ± 0.21 ^a^	92.46 ± 0.26 ^a^	91.48 ± 0.32 ^b^
IC_50_	14.14 ± 0.41 ^b^	14.35 ± 0.22 ^ab^	14.61 ± 0.28 ^a^

Values were reported as means of replicates ± SD. Values in the same row with different letters have significant differences (*p* < 0.05); * P, picual; T, tofahi; S, shemlali.

**Table 5:** The hemolytic activity (%).

Concentration (μg/mL)	HRBC Membrane Hemolysis (%)		
	P *	T *	S *
50	12.00 ± 0.15 ^a^	11.58 ± 0.10 ^a^	12.44 ± 0.13 ^a^
100	8.51 ± 0.22 ^b^	10.06 ± 0.19 ^a^	7.70 ± 0.16 ^b^
200	4.21 ± 0.14 ^b^	3.90 ± 0.15 ^b^	6.00 ± 0.19 ^a^
500	3.40 ± 0.17 ^b^	3.80 ± 0.11 ^a^	3.80 ± 0.13 ^a^

Values were reported as means of replicates ± SD. Values in the same row with different letters have significant differences (*p* < 0.05; * P, picual; T, tofahi; S, shemlali).

**Table 6:** Level of glucose and HbA1c in diabetic rats.

Group	Glucose (mg/dL)	HbA1_C_ (%)
Negative control (NC)	112.00 ± 3.05 ^b^	4.40 ± 0.10 ^b^
Positive control (PC)	340.00 ± 6.82 ^a^	13.80 ± 0.20 ^a^
Metformin	154.00 ± 4.10 ^b^	4.80 ± 0.10 ^b^
OLE	174.33 ± 7.14 ^b^	4.80 ± 0.10 ^b^
Metformin + OLE	121.67 ± 5.49 ^b^	4.70 ± 0.10 ^b^

Each reported value is the mean ± SD of replicates; Means in the same column followed by different letters are significantly different (*p* ≤ 0.05).

**Table 7:** Effect of olive leaf extracts on the lipid profile of streptozotocin-induced diabetic rats.

Group	Cholesterol (mg/dL)	Normal	Triglycerides (mg/dL)	Normal	HDL (mg/dL)	Normal	LDL (mg/dL)	Normal
NC	60.00 ± 4.50 ^ab^	<200 mg/dL	106.33 ± 1.53 ^c^	<150 mg/dL	54.00 ± 3.21 ^a^	>40 mg/dL	38.69 ± 0.07 ^b^	<100 mg/dL
PC	174.00 ± 4.58 ^a^		152.00 ± 1.73 ^a^		40.00 ± 3.06 ^a^		120.15 ± 0.33 ^a^	
OLE *	52.00 ± 1.73 ^b^		118.33 ± 2.89 ^b^		48.00 ± 5.51 ^a^		60.36 ± 0.33 ^b^	
Metformin	72.33 ± 5.33 ^a^		109.00 ± 2.65 ^c^		48.33 ± 2.96 ^a^		56.33 ± 0.33 ^b^	
Metformin + OLE	49.33 ± 1.45 ^b^		109.00 ± 3.60 ^c^		52.33 ± 4.91 ^a^		51.46 ± 0.33 ^b^	

Each reported value is the mean ± SD replicates; means in the same column followed by different letters are significantly different (*p* ≤ 0.05); OLE *: olive leaf extract, NC: negative control; PC: positive control.

## Data Availability

The data presented in this study are available on request from the corresponding author.
